# The Whistleblower's Dilemma in Young Children: When Loyalty Trumps Other Moral Concerns

**DOI:** 10.3389/fpsyg.2018.00250

**Published:** 2018-03-01

**Authors:** Antonia Misch, Harriet Over, Malinda Carpenter

**Affiliations:** ^1^Department of Developmental and Comparative Psychology, Max Planck Institute for Evolutionary Anthropology, Leipzig, Germany; ^2^Department of Psychology, University of York, York, United Kingdom

**Keywords:** intergroup cognition, group loyalty, morality, whistleblowing, social cognition

## Abstract

When a group engages in immoral behavior, group members face the whistleblower's dilemma: the conflict between remaining loyal to the group and standing up for other moral concerns. This study examines the developmental origins of this dilemma by investigating 5-year-olds' whistleblowing on their in- vs. outgroup members' moral transgression. Children (*n* = 96) watched puppets representing their ingroup vs. outgroup members commit either a mild or a severe transgression. After the mild transgression, children tattled on both groups equally often. After the severe transgression, however, they were significantly less likely to blow the whistle on their ingroup than on the outgroup. These results suggest that children have a strong tendency to act on their moral concerns, but can adjust their behavior according to their group's need: When much is at stake for the ingroup (i.e., after a severe moral transgression), children's behavior is more likely to be guided by loyalty.

## Introduction

During recent years, high profile cases of whistleblowing have garnered enormous public attention and caused controversy in politics and the international media. For example, recently, the former CIA contractor Edward Snowden, who revealed top-secret information about surveillance programs run by the US National Security Agency, was extensively both reviled and lauded in equal measure for being a whistleblower. Whistleblowing is the disclosure of one's own group's transgressions with the intention of stopping the group's wrongdoing, which necessarily involves an act of disloyalty against the group (see Jubb, 1999). Whistleblowers thus experience a dilemma in which they have to decide whether to act on their feelings of group loyalty or on other moral principles (Waytz et al., 2013). According to Haidt and colleagues, loyalty is one of five moral foundations (Haidt and Joseph, 2007) and requires preferential treatment for members of one's own group. In contrast, other moral concerns, such as fairness and care, demand equal treatment for all (Haidt and Graham, 2007). Thus, loyalty can involve sacrificing other moral principles to protect the group, while whistleblowing involves privileging these other moral concerns over loyalty. The consequences of whistleblowing for both the group and the whistleblower can often be severe. The group may be punished externally, and the whistleblower may be punished by the group as a traitor, and maybe even excluded or banned.

Surprisingly, the conditions under which people decide whether to blow the whistle on their group have not been extensively investigated. Research with adults has examined the effects of factors such as the interests of the group and role responsibility (Trevino and Victor, 1992), or monetary incentives and legal protections (Oh and Teo, 2010). Other studies have focused on whistleblowing in interpersonal rather than group contexts (e.g., Gino and Bazerman, 2009; Bocchiaro et al., 2012; Waytz et al., 2013). Only a small amount of research has directly investigated the effects of morality and loyalty concerns on whistleblowing. A set of studies conducted by Waytz et al. (2013) suggests that participants' willingness to blow the whistle on another person is predicted by their endorsement of fairness over loyalty concerns. They also found that participants' willingness to blow the whistle decreases with closeness between the participant and the transgressor. It is not yet clear, however, what happens when loyalty and other moral concerns are directly pitted against each other in a group context. Furthermore, a common feature of previous research is that it has assessed participants' predictions of how they might act if faced with this dilemma. But evidence suggests that participants' predictions can diverge from their actual behavior. For example, in a study conducted by Bocchiaro et al. (2012), a large majority of participants predicted that they would blow the whistle on an unethical request, but only a small minority actually did so when put to the test, stressing the importance of investigating the whistleblowing dilemma in a behavioral set-up.

Developmental research has shown that both components of the whistleblower's dilemma, feelings of group loyalty and other moral concerns, are present early in childhood. At least by 5 years of age, children clearly value loyalty to the group: They favor loyal over disloyal group members (e.g., Abrams et al., 2003, 2008; Misch et al., 2014). They also show loyal behavior themselves, even when it is costly for them to do so (Misch et al., 2016). Young children are also sensitive to other basic moral principles. For example, from the age of 3 years, children actively intervene in moral transgressions in which a third party has been harmed (Rossano et al., 2011; Vaish et al., 2011), give more resources to an individual who behaved in a morally good way (Kenward and Dahl, 2011), and avoid helping people with harmful intentions (Vaish et al., 2010). They are also concerned with fairness, for example they prefer a fair to an unfair distributor in a third party context (e.g., Shaw et al., 2012).

However, to our knowledge, no study has directly investigated the conflict between loyalty and other moral considerations in an intergroup context in young children. The few studies that have investigated the related issue of the interplay between ingroup favoritism^1^ and fairness in children have found mixed results. DeJesus et al. (2014) found that in a third-party context, at least from 6 years of age, children expect others to favor their ingroup, but evaluate fair distributions as nicer. However, when evaluating their own ingroup members' resource distributions between groups, Cooley and Killen (2015) found that 3.5- to 6-year-old children value fairness over group considerations, whereas Jordan et al. (2014) found that 6- and 8-year-old children tended to decide whether to punish unfair distributors based on group membership, and in doing so, sacrificed moral considerations that demand equal treatment for all (Rhodes and Chalik, 2013).

The studies that have come closest to investigating the conflict between loyalty and other moral concerns are studies on so-called “blue lies”—the opposite of whistleblowing—that is, lies that are told to protect someone else. Several studies have investigated children's evaluations of blue lies in story vignettes and found that with age, children evaluate blue lies to cover up the ingroup's transgression more positively (e.g., Sweet et al., 2010; Lau et al., 2013; Chiu Loke et al., 2014; Fu et al., 2016). To our knowledge, only one behavioral study has directly focused on children's blue lies by asking participants to report their own group's wrongdoing. Fu et al. (2008) tempted class groups of 7- to 11-year-old Chinese children to cheat in a competition by allocating more expert players to their team than were allowed. Afterwards, an uninvolved experimenter asked children in a confidential one-to-one situation whether their team really played by the rules. The majority of children confessed their team's transgression and thus acted according to their moral considerations rather than their feelings of loyalty. However, this study did not include an outgroup comparison so it is not known whether children would have been even less likely to lie for an outgroup. It thus still remains open how children would weigh moral and loyalty concerns when deciding what to do about an ingroup vs. an outgroup member's transgression.

A promising approach to study this conflict is to look at children's tattling behavior. The terms tattling and whistleblowing are often used interchangeably (see e.g., Waytz et al., 2013), but one important distinction will be made here: While tattling can be used rather generally and independently of group membership or affiliations (see e.g., Ingram and Bering, 2010), whistleblowing refers specifically to tattling about one's own organization or group (e.g., Jubb, 1999). For children, tattling is a frequent and natural way of dealing with others' transgressions and misbehavior. Young children do not perceive tattling as negative and thus frequently tattle on peers in school (Ingram and Bering, 2010), on their siblings (Den Bak and Ross, 1996), on puppets in experimental settings (Vaish et al., 2011; Schmidt et al., 2012), and even on adults' transgressions (Heyman et al., 2016).

To investigate the origins of the whistleblower's dilemma in young children, we thus study children's tattling behavior. Children observed either ingroup or outgroup members commit a moral transgression. Afterwards, an uninvolved experimenter entered the room and gave children the chance to spontaneously tattle before asking more direct questions. We expected that children would be more likely to tattle on the outgroup's than on the ingroup's transgression, because previous developmental research has shown that young children are loyal to their groups (Misch et al., 2016) and research with adults has shown that the closeness of one's relationship to the transgressor is negatively correlated with the likeliness to blow the whistle on him/her (Waytz et al., 2013). We chose to test 5-year-old children because this is the earliest age at which clear evidence exists that children both value loyalty to the group (Misch et al., 2014, 2016) and are concerned about moral transgressions (Blake and Harris, 2009; Rossano et al., 2011; Vaish et al., 2011).

Additionally, to investigate the conflict between loyalty and morality more deeply, we were also interested in the impact of the severity of the transgression. More specifically, we wished to examine whether and how loyalty and the severity of the moral violations would interact. Results from previous studies looking at children's evaluations of and reactions to different types of transgressions are mixed. One line of research has found that 4- to 7-year-old children endorse tattling on both major and minor transgressions equally, and only from around 8–9 years endorse tattling on major more than on minor transgressions (Lyon et al., 2010; Loke et al., 2011; Chiu Loke et al., 2014; Heyman et al., 2016). However, in these studies children were simply asked to evaluate or predict vignette story characters' tattling behavior. Behavioral studies that have investigated children's own behavior following different types of transgressions have found that by 3 years of age, children differentiate between severe moral transgressions and more minor conventional violations in that they protest more strongly when someone destroys the possession of another person compared to when someone plays a game incorrectly (Schmidt et al., 2012).

In the current study, the transgression was implemented in the form of a theft. Previous research has shown that from around 3 years of age, children understand the violation of property rights and protest against this (Rossano et al., 2011). At least by age 5, they understand the illegitimate nature of stealing (Blake and Harris, 2009). An advantage of using this type of transgression is that it allowed for a quantitative manipulation of severity: Children in the mild transgression condition observed two puppets take only a little bit of someone's possession (i.e., 1 out of 10 gemstones), while children in the severe transgression condition observed these puppets take nearly all of that resource (i.e., 9 out of 10 gemstones). For children for whom these two puppets were outgroup members (outgroup condition) we expected generally high levels of tattling in both transgression conditions (although they might tattle more in the severe transgression condition). Children should not feel any loyalty to the outgroup members, and therefore should act according to their moral considerations and, consequently, tattle. For children for whom the transgressors were ingroup members (ingroup condition), observing the mild vs. severe transgression should also elicit mild vs. severe moral considerations; however in this case these considerations should conflict with loyalty considerations. We expected that children's feelings of group loyalty would make it more difficult for them to blow the whistle on their ingroup members. There were two different possible ways in which the severity of the transgression might influence their behavior.

The first possibility was that in the mild condition, compared to their feelings of loyalty, children's moral considerations should be relatively low, and consequently children might act according to their feelings of loyalty and keep quiet about their group's transgression. After the severe transgression, however, moral considerations should outweigh feelings of loyalty, and thus children might act on their moral considerations and blow the whistle on their group.

The social psychological literature with adults suggests a second possibility. According to a nonabandonment norm, group members should stick to their group in all circumstances (Zdaniuk and Levine, 2001), but especially in situations in which it is needed most (e.g., because the group is under threat; see Ellemers et al., 2002; Van Vugt and Hart, 2004). Indeed, some evidence supports the notion that threat to the group increases group cohesion or ingroup bias (Turner et al., 1984; Hunter et al., 2005), and that after undergoing negative experiences, group members feel more fused with each other (e.g., Jong et al., 2015) and show more pro-group behavior (Swann et al., 2014). If this is the case, then children should keep quiet after their own group's severe transgression, as otherwise the group members would have to face punishment or other negative consequences. After a mild transgression, in contrast, potential negative consequences should be relatively minor and not harm the ingroup much; therefore children could act according to their moral considerations and blow the whistle.

## Methods

### Participants

Participants were 96 5-year-old children (48 girls and 48 boys, age range 5 years; 27 days – 5 years; 9 months, 9 days; *M* = 5 years, 6 months). The number of participants (24 per condition) was specified in advance based on previous research (Misch et al., 2016). Twenty-two additional children were tested but excluded for failing one of the critical control questions that tested whether they understood the procedure (i.e., failing to correctly say which group they were in [1], failing to correctly say which group the transgressors were in [4], or failing to remember whether one vs. many gemstones were taken away [8]), or for experimenter error (5), not responding at all (1), leaving the room during the procedure (2), or naming one of the transgressors after herself (1).^2^

Children were recruited and tested in their daycare centers in a mid-sized city in Germany. The test session took approximately 20 min. No SES or ethnicity data were collected, but approximately 98% of the population from which the sample was drawn are native German. The study was developed and conducted in accordance with ethical guidelines and was approved by the institution's ethics committee (Max Planck Institute for Evolutionary Anthropology Child Subjects Committee).

### Materials

**Figure 1:**
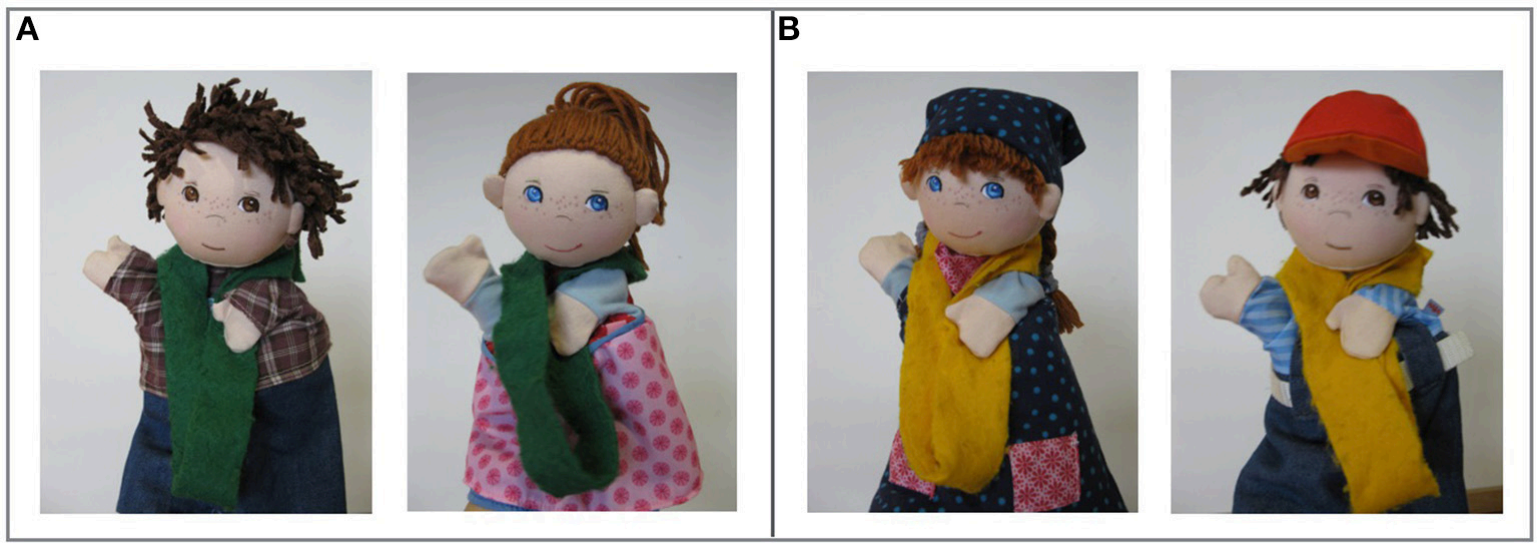
The puppets used in this study. **(A)** The transgressors played by E1 (here wearing green group markers), **(B)** The puppets played by E2 (here wearing yellow group markers).

We used puppets as in- and out-group members because previous work has shown that children are willing to tattle on puppets' transgressions (e.g., Vaish et al., 2011). Children were tested by three female experimenters: a moderator (M) and two puppeteers (E1 and E2). Each puppeteer played one female and one male hand puppet (see Figure 1). The two puppets played by E1 were the transgressors. In the ingroup condition the child was allocated to the same group as the transgressors; in the outgroup condition the child was allocated to the other group.

**Figure 2:**
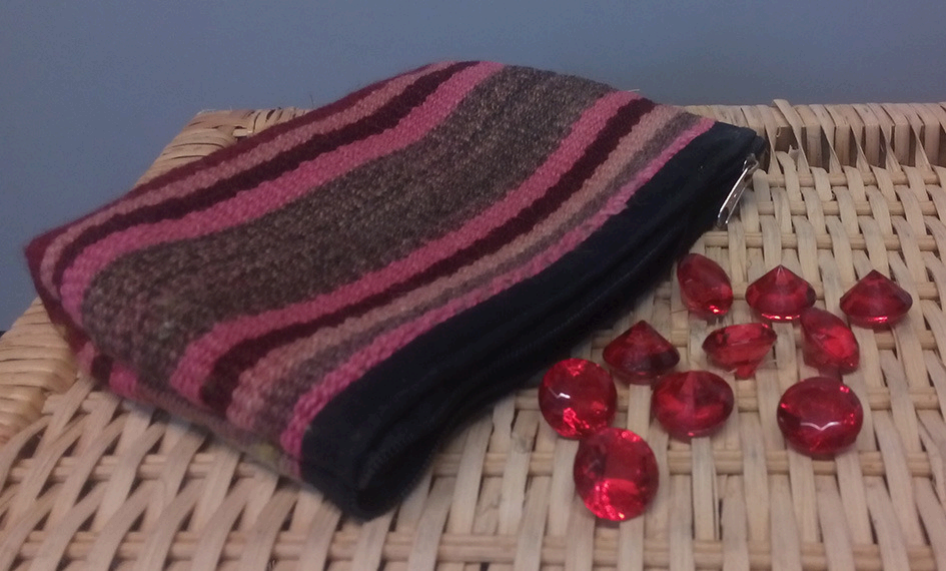
The ten gemstones used as spoils.

A set of green and yellow scarves (two puppet-sized scarves and a child-sized scarf in each color; see Figure 1) were stored in a box with a lid. Ten fake red gemstones were used as spoils (see Figure 2). They were hidden in a small purse located on a box on the left side of the room (approximately 2 m away from the door).

There was a low cardboard barrier (30 cm in height) on the other side of the room. Thirty large marbles and a marble bag were used to keep children occupied and in place before and during the transgression, and a marble run was used for the preference test at the end.

### Design and counterbalancing

Children were tested in a 2 × 2 between-subjects design. We manipulated the transgressors' group membership and the transgression type: Transgressors were either in the child's in- or out-group, and took either a little (only one out of 10 gemstones in the mild condition) or a lot (nine out of 10 gemstones in the severe condition).

Across children, we counterbalanced the color of the child's group (so that half of the children in each condition were in the yellow group, and the other half were in the green group) and the color of the transgressors' group (so that half of the time they were in the yellow group, and half of the time they were in the green group).

### Procedure

Children were picked up from their classroom individually by all three experimenters. At the start of the procedure, there was a brief warm-up phase during which children became acquainted with the experimenters and the four puppets that would later be allocated to groups. First, the moderator (M) introduced the child to the puppets and then asked the puppets to introduce themselves. Following this, M asked the child and each of the puppets two questions to engage them in a brief conversation (e.g., about what they had had for breakfast, or which parent dropped them off at the daycare). This was done in order to make the child feel comfortable in the situation and to establish that the puppets should be treated as if they were real individuals around the same age as the child.

#### Group allocation

After the warm-up, M allocated the child and the four puppets to groups. She did this by saying, “Today, we need two different groups. We will have a yellow group and a green group. First of all, we need to know which group everyone belongs to.” M then picked up the box and explained that in this box there were yellow and green scarves, and that she would now pull out one scarf for each of them, thereby finding out which group they belonged to. Then, one by one, she allocated each of the puppets and the child into groups by apparently randomly drawing yellow and green scarves out of the box and placing them on each individual's neck. Group allocation always started with one of the child's ingroup puppets, then proceeded to an outgroup puppet, then to the child, the other outgroup puppet, and finally the other ingroup puppet.

#### Transgression

After the group allocation, M said that next they would need the marbles that were lying on the floor behind the low barrier in one corner of the room. She noticed that the marble bag was missing and asked the child to come with her to look for the bag outside of the room. This was an excuse so that E1 and E2 could leave the room unseen and wait in an adjacent room. When M and the child returned with the marble bag, M pretended to be surprised that the others were missing and asked the child to put all the marbles into the bag while she looked for the others outside. The task of putting the marbles into the bag was given to children so that they would be occupied with a simple activity on one side of the room, but would still be attentive enough to observe the transgression. While the child was busy picking up the marbles, the two puppets played by E1 entered the room. Depending on condition, they were either in the same group as the child (ingroup condition) or in the other group (outgroup condition). They recognized and greeted the child very briefly, before turning to each other and ignoring the child. The male puppet then said, “Look, there is the purse! Maybe there are gemstones in it again, and we could take some again!” To make sure that children understood that the puppets were not entitled to take the gemstones, the other puppet was skeptical and pointed out that the gemstones did not belong to them. In order to convey the idea that this was something this group did regularly, the first puppet said “But we are members of the yellow/green group, and the yellow/green group always does it like that!” The female puppet then replied, “Ok, then let's have a look. But let's be quick and quiet, so that no one will catch us!” They then opened the purse and admired the gemstones. Depending on the condition, they took either one (mild transgression) or nine of the ten gemstones (severe transgression). In the mild condition they said to each other, “Let's take only a little bit, only one gemstone. There are still many left, certainly no one will notice!” In the severe condition they said, “Let's take a lot of them, nearly all the gemstones. There is still one left, certainly no one will notice!” After they put the gemstone(s) into their purse, M called them from the outside, “[Transgressor puppets' names], where are you?” The puppets replied to M, “We are coming,” and then said to themselves, “Let's leave quickly, so that no one will catch us!” Finally, before leaving the room, they asked the child to wait inside.

#### Tattling opportunity

Then, M entered the room and gave the child the chance to tattle. In order to assess how quickly and spontaneously children tattled, she used a stepwise, ramping-up procedure with a 5-s pause in between each step to give children time to tattle. She first started with very general comments (Step 0: “I'm back” and Step 1: “Is everything okay?”), and then gave some hints that something was amiss while looking at the bag (Step 2: “What is going on here?” and Step 3: “What did I miss?”). She then asked more directly about the bag (Step 4: “There is a bag. Someone must have forgotten it…” and Step 5: “The zipper is open. Maybe someone took something out?”). In the final step she finally suspected the puppets directly (Step 6: “I think I just saw [names of transgressor puppets] leave, maybe they took something?”). If the child did not respond at all during a given 5-s response period, or only said something unrelated (e.g., just talked about the marbles that they had picked up), M moved on to the next step. For children's statements to qualify as tattling, they had to make it clear that someone had taken something away (for step 6 it was sufficient if they confirmed M's suspicion by saying “yes”). If children only gave a hint of this, M further encouraged them by saying “Uh huh, tell me!” If children correctly described what had happened but failed to name the transgressor(s), M asked “And who?” Following that, children had another 5 s until, if needed, M moved on to the next step. To minimize social pressure on children, M looked only briefly at them and then continued to inspect the scene. Thus, children were free to remain silent.

#### Post-test measures and resolution

To explore the motivation underlying children's behavior, we asked them some post-test questions about their justification for and evaluation of the transgression, their judgment of the transgressors, their own accountability (only for tattlers), their loyalty, and their group preference. Because these questions were exploratory, we did not push children to answer if they did not respond. As a consequence the number of *no answer* responses was relatively high and the results should be taken with some caution. Furthermore, grouping children depending on their tattling behavior led to small and uneven sample sizes in the different cells. Thus, for most of the measures, statistical analysis was not appropriate; therefore we report these results in the Supplementary Material.

##### Memory questions

After the first set of post-test questions (but before the loyalty question), in order to make sure that children had followed and understood the procedure, M asked children three memory questions: “Which group did the two who took the stones belong to?,” “How many stones did they take, many or just a few?,” and “Which group do you belong to?”

##### Resolution of the situation

After M's loyalty question, before the preference test, the two transgressor puppets re-entered the room. They were clearly upset by their wrongdoing, confessed everything to M, and apologized. M explained that taking away others' belongings is not okay and made them promise never to do anything like this again. Then the other two puppets came back and everyone played together with a marble run. Finally, children were thanked and taken back to their classroom.

### Coding and reliability

Our main interest was in whether children tattled on the puppets' transgression or not (saying, e.g., “They took the gemstones” or “I saw that they stole something”). Children's statements were coded as tattling if they made it clear that someone (e.g., “they,” “the puppets,” “the two,” or using their names) had taken something away (e.g., “They took something,” “They swiped the stones”). Only for Step 6 was it sufficient if they clearly confirmed M's suspicion by saying “yes”).

In addition, for those children who tattled, we also investigated how quickly and spontaneously they tattled. For this analysis, children received a score between 0 and 7, corresponding to the step at which they tattled (e.g., they received 1 if they tattled at step 1, or 6 if they tattled at step 6). If they tattled before M's first hint, they received a 0, and if they did not tattle at all, they received a score of 7.

The main coding was done by the first author. To assess inter-rater reliability, an independent coder who was unaware of the hypotheses of the study coded a random sample of 25% of children for both measures together from the videos. Reliability (Cohen's weighted kappa) was perfect with κ = 1.00.

## Results

All statistical analyses were performed using R (R Core Team, 2014) version 3.2.0. Significance of the models was tested using both likelihood ratio tests (LRT), by comparing the fit of the full model with that of the respective reduced models, and the *p*-values provided by the final model.

A preliminary analysis revealed no effects of children's gender or color group on the main results regarding children's tattling (General Linear Model, full-null model comparison, *p* > 0.25). Therefore, we collapsed across these variables and do not consider them further.

**Figure 3:**
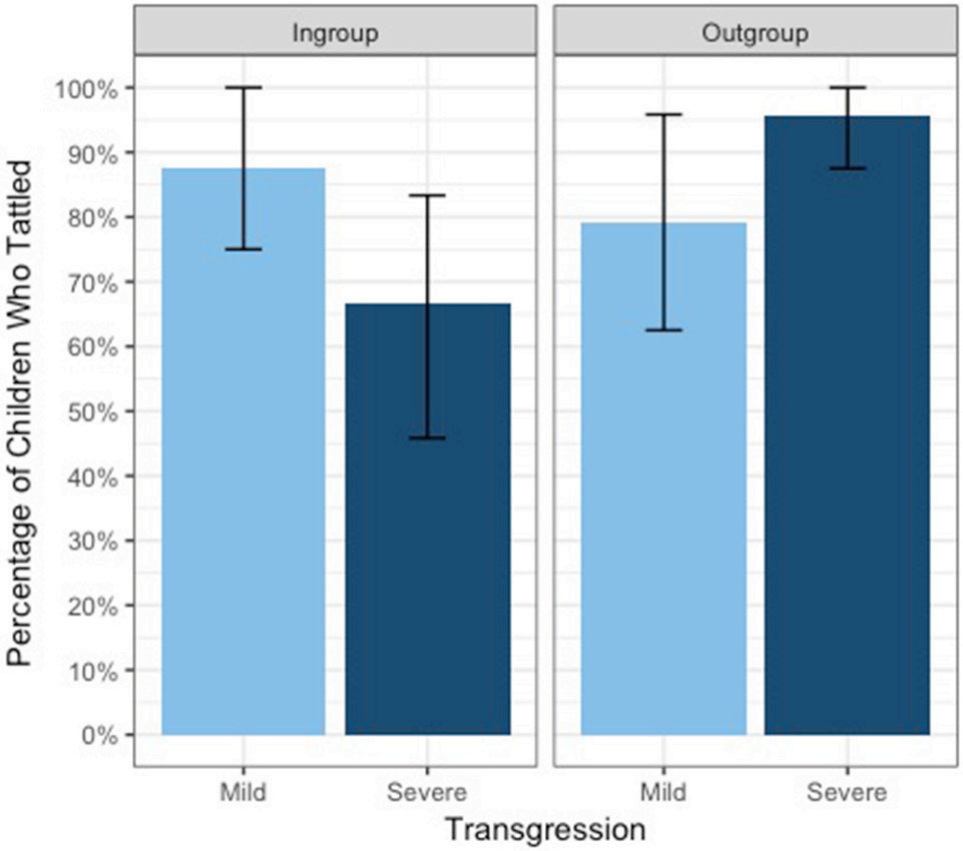
Percentage of children who tattled, by group membership and transgression type, with bootstrapped 95% confidence intervals.

Our main interest was in how many children tattled about the puppets' transgression at any point during the test phase. Overall, across all four conditions, the majority of children tattled (82.3%), suggesting a general concern for harm. Figure 3 depicts the percentage of children who tattled in each condition for each transgression type.

A GLM was run with group membership and transgression type as predictors, and the binomial measure of tattling (yes or no) as response variable. The full model differed significantly from the null model [χ^2^(3) = 8.14, *p* = 0.043] and revealed a significant interaction between group membership and transgression type [*Estimate* = 3.05, *SE* = 1.36, χ^2^(1) = 6.26, *p* = 0.012, Nagelkerke's *R*^2^ = 0.11]. Post-hoc tests revealed that children in the ingroup condition were significantly less likely to tattle on a severe transgression than were children in the outgroup condition (Fisher's exact test, *p* = 0.023, risk ratio = 2.17); all other pairwise comparisons were non-significant (Fisher's exact tests, all *p'*s > 0.16).

**Figure 4:**
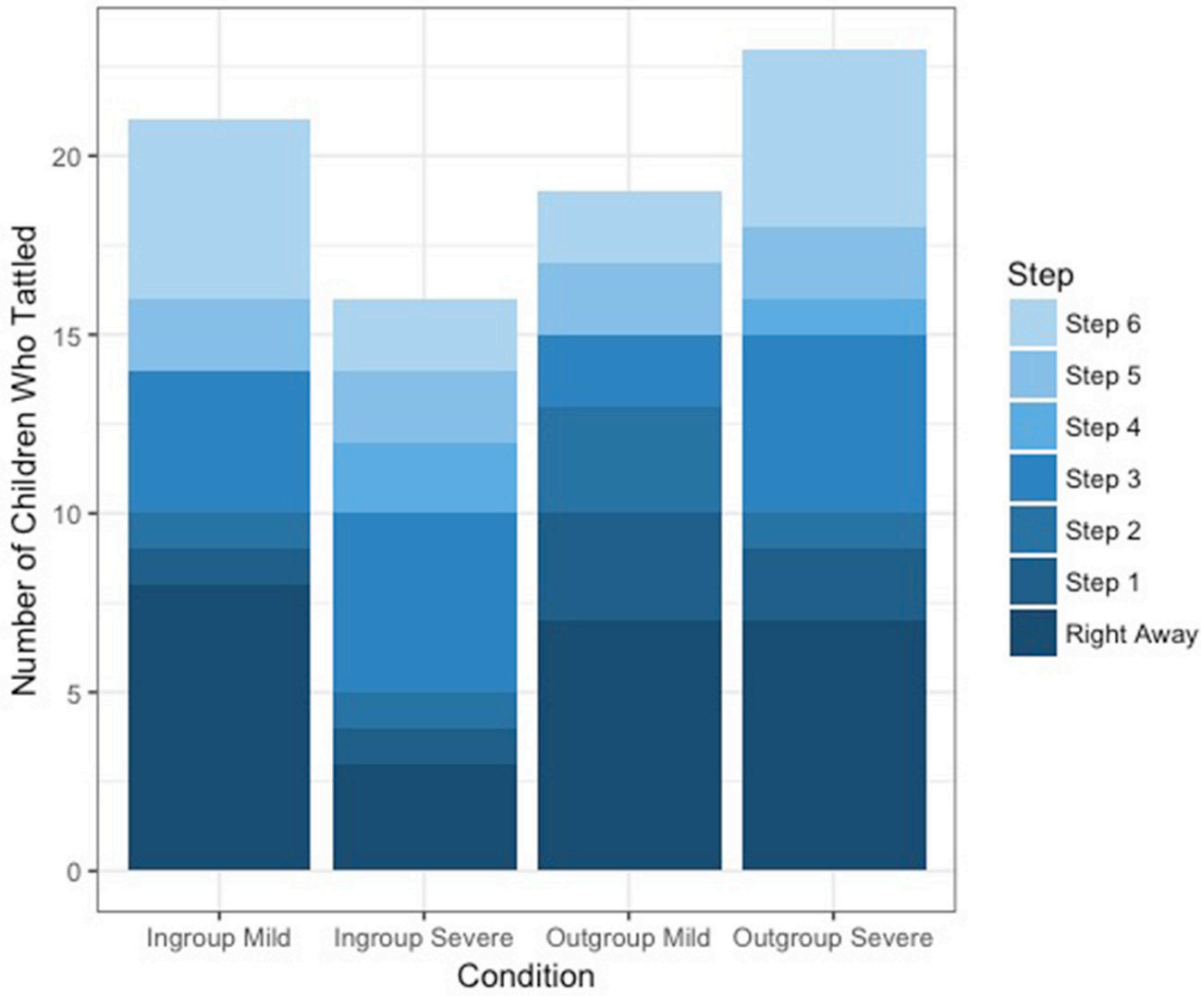
Number of children who tattled at each step, by condition.

To investigate whether the conditions had an effect on how quickly children tattled, we ran a GLM with Poisson distribution only on children who had tattled at some point (*n* = 79), with children's tattling score (0–6) as the response variable. The full model did not differ from the null model (*p* = 0.21), indicating that the conditions had no significant effect on how quickly children tattled (see Figure 4).

## Discussion

The aim of this study was to examine the whistleblower's dilemma: the conflict between feelings of loyalty and other moral concerns. This was done by looking at children's willingness to blow the whistle on their in- vs. out-group members' mild vs. severe transgression. An interesting pattern of results emerged: Rather than simply tattling more on outgroup members across the board, children showed a complex weighting of loyalty and moral considerations. After the mild transgression, children acted on their moral considerations: They tattled on both groups at equally high rates. After the severe transgression, however, they were significantly less likely to blow the whistle on their ingroup than on the outgroup, suggesting that children's feelings of loyalty to the group sometimes outweighed other moral considerations. Consistent with the idea of a nonabandonment norm, these results support the notion that group loyalty becomes most important when much is at stake for the group, that is, that one should show the strongest loyalty when the group is under threat (Ellemers et al., 2002; Van Vugt and Hart, 2004). These results therefore suggest that young children are already capable of flexibly weighing moral and loyalty considerations and, in some cases, are willing to sacrifice their moral principles for the sake of their group.

There are a number of possible motivations that could have been underlying children's loyal behavior. We aimed to investigate these with the questions we asked children following the tattling phase. Unfortunately, these findings were underpowered due to low response rates (see Supplementary Material). Still, some of our and others' findings can shed light on the possible underlying motivations. For example, it is possible that children generally perceive transgressions of their ingroup members as less severe than transgressions of their outgroup members and that this led them to blow the whistle less often in the ingroup condition. Previous research has shown that children are more forgiving and forgetful when it comes to the negative behavior of their ingroup members (Corenblum, 2003; Dunham et al., 2011). However, in the mild condition of the current study children were equally likely to blow the whistle on their in- and out-group members, suggesting that this factor alone cannot explain the observed results. Another possibility is that children might have wanted to protect their ingroup from the potential negative consequences (e.g., punishment) of their whistleblowing and/or avoid being punished themselves. Previous work has shown that children feel responsible for their group members' negative actions (Over et al., 2016), and consequently children's feelings of shame or embarrassment about their group members' transgression might have decreased their whistleblowing in the ingroup severe condition. Relatedly, some children might have been shocked about their group's transgression and thus too preoccupied to speak out about it. Future research should thus investigate the role of moral emotions such as guilt and embarrassment, and also the fear of negative consequences in the context of loyal behavior. Another potential reason for children's increased loyalty after the severe transgression is the fact that the group was now in possession of the stolen goods. Previous research has shown that children prefer wealthy over less wealthy groups (Horwitz et al., 2014) and show more loyalty to groups that are of high status (e.g., Nesdale and Flesser, 2001). However, children's justifications for why they wanted to stay in or leave their group suggest that this was not the reason for their choice in this situation: No child ever justified their choice to stay with or join the transgressors' group by mentioning the group's wealth, higher status, or the possession of the stones more generally, while the transgression was a common reason for joining or not leaving the non-transgressors' group.

In future research, it would be informative to investigate in which situations children are willing to override their moral concerns in order to remain loyal. It would be interesting to look at children's loyalty after even more different types of transgressions, including a wider range of severity and different kinds of moral violations. Also, if children were asked to choose between internal within-group protest (i.e., scolding and correcting ingroup members privately) and external tattling (i.e., telling someone outside the group more publicly), would their choice depend on their group membership?

In summary, our findings suggest that both loyalty and other moral considerations guide 5-year-old children's behavior. When moral concerns are relatively low, children act freely on them by tattling on the outgroup and even blowing the whistle on their own group. In contrast, when moral concerns increase, children's behavior is guided by their loyalty: They tattle freely on their outgroup, but are less likely to blow the whistle on their own group. Thus, already by 5 years of age, children consider both loyalty and other moral concerns together, and adapt their behavior flexibly. Even though they clearly understood the negative nature of the transgression, they were willing to sacrifice their personal moral concerns for the sake of their group. This is an interesting finding, given the fact that from very early on, children show a strong appreciation for key moral domains such as care and fairness (e.g., Hamlin et al., 2007; Hamlin and Wynn, 2011; Vaish et al., 2011), while robust preferences for minimal ingroups and clear loyal behavior do not appear much before the age of five (Dunham et al., 2011; Misch et al., 2016). Thus, right around the time that loyalty to the group first appears in ontogeny, it can already have a dark side, overriding other moral concerns. This can lead to rather undesirable behavior on the one hand, for example when it results in concealing moral transgressions of the ingroup. However, from the perspective of the group, it may be seen as desirable in that it helps ensure from early on that group members are trustworthy and protective of their group and thus that they can be counted on when most needed.

## Author contributions

All authors developed the study concept and design. Testing, data collection, and data analysis were performed by AM, who also drafted a first manuscript. MC and HO provided critical revisions. All authors approved the final version of the manuscript for submission.

### Conflict of interest statement

The authors declare that the research was conducted in the absence of any commercial or financial relationships that could be construed as a potential conflict of interest.

## Footnotes

^1^Note that loyalty to the group is more than simple ingroup preference, in that it entails a sense of commitment and often the willingness to sacrifice personal benefits for the sake of the group (see Brewer and Silver, 2000, p. 162).

^2^For 21 of these excluded children it was still possible to obtain a tattling score. Adding them to the main analysis did not change the results [full-null model comparison: $\chi_{\left(3\right)}^{2}$ = 8.04, *p* = 0.045; interaction between group membership and transgression type: *Estimate* = 2.84, *SE* = 1.14, $\chi_{\left(1\right)}^{2}$ = 7.08, *p* < 0.01].
