# Supplementary material for: The Whistleblower's Dilemma in Young Children: When Loyalty Trumps Other Moral Concerns
Source: Front Psychol. 2018 Mar 1;9:250. doi: 10.3389/fpsyg.2018.00250 (PMC5839002; doi:10.3389/fpsyg.2018.00250)
Supplement: Supplementary file 1 [file Table1.pdf]

THE WHISTLEBLOWING DILEMMA IN YOUNG CHILDREN

Supplementary Material

Procedure

To help understand the motivations behind children's responses, we asked children some exploratory questions after the main test phase. To ensure that all children felt comfortable talking about the transgression, at this point M confirmed her suspicion that the two transgressors took something, expressed at Step 6, by saying aloud to herself, "That is what must have happened."

Transgression justification. To see whether children would come up with excuses for the transgressors and, if so, to check whether the excuses would differ between conditions, children were asked why they thought the puppets took the gemstones.

Judgments. Then, to see whether children understood the transgression as morally wrong, and to examine whether they would judge this differently between conditions, M asked them to judge the severity of transgression, asking, "Do you think what they did was good, okay, not so good, or bad?" and "Why do you think this was [children's response]?"

Punishment. After that, to see whether children thought there should be consequences for the transgression, and whether their perception of these would differ between conditions, children were asked, "What should we do with them when they come back?" and "Should they be punished?" If children responded with "yes" to the latter question, they were then asked, "How should they be punished?"

Accountability. To see whether children were aware that tattling might have negative consequences for themselves if the transgressors found out that children had tattled, all children who had tattled were asked, "Would it be ok if they [the transgressors] knew that you told me about this?"

Exit choice and exit choice justification. After that, children were given a chance to leave their group: M discovered one additional group marker from the child's outgroup, and asked children whether they wanted to change groups or wanted to stay in their group, and why they decided to stay or leave.

Preference test. All four puppets, the child, and M sat together around the marble run for a final preference test. The child was given the first marble to put down the marble run. Then, M asked the child to distribute four more marbles, one by one, to the puppets.

After that, all the puppets and the child engaged in free play with the marble run.

Coding and Reliability

Along with exploring children's responses to the post-test questions, we also took a closer look at children's tattling responses. For the post-test questions, if

children did not respond, shrugged, replied with “I don’t know,” or gave a response that was unrelated to the question, their answer was coded as *no answer*. Children were not prompted again in these cases, as these questions only aimed at providing additional information. An independent, naïve coder coded a random sample of 25% of children’s transcribed responses.

Tattling words. First, we looked at what words children used when tattling about the puppets’ transgression. We were interested in whether children would use neutral vs. more negative words when tattling, as this could reflect their evaluation of the severity of the transgression. Thus, for all the children who tattled, we categorized the terms they used into *neutral* words, which described the event without evaluating it (e.g., “leaving only one,” “taking out,” “putting into her pocket”) and more *negative* words (e.g., “stealing,” “taking away,” “swiping”). Reliability (Cohen’s weighted kappa) was good with $\kappa = 0.69$.

Transgression justifications. After the main test, the moderator asked children why they thought the puppets had taken the gemstones. To investigate whether children would downplay vs. emphasize the transgression, their responses were coded as *positive/mitigating* (e.g., “because the stones were so pretty,” “the stones were just lying around,” “they were just curious”), *negative/aggravating* (e.g., “because they wanted to steal them,” “because they are thieves”), and *neutral/other* (e.g., “they wanted to,” “because they are in the yellow group”). Reliability was excellent with $\kappa = 1$.

Judgments and judgment justifications. Children’s judgments of the puppets’ transgression were coded as scores on a Likert scale ranging from 1 (“good”) to 4 (“bad”). Children were also asked to justify their judgments. These justifications were coded into categories referring to *moral* (focusing on the harm that was done, e.g., “now only one is left,” “if it belongs to someone, he will be sad when he returns”), *normative* (referring to norms, rules, permission, and ownership rights, e.g., “one shouldn’t do this,” “they didn’t ask”), and *other* reasons (e.g., “because they were red,” “I didn’t think they would do it”). Reliability was excellent for children’s judgments (Cohen’s weighted kappa) with $\kappa = .94$, and fair for children’s judgment justifications (Cohen’s kappa) with $\kappa = 56$.

Punishment. We coded whether children thought that the transgressors should be punished (“yes”) or not (“no”), and, if so, what kind of punishment they proposed in response to the other two questions (i.e., “What should we do with them?” and “How should they be punished?”). Since there was considerable overlap between children’s responses to these similar questions, we combined their responses to these questions and, if they differed, counted the most severe form of punishment they suggested. Response categories were *lecturing* (verbal forms of punishment and lecturing, e.g., “scold them,” “tell them that this is not okay”), *compensate* (requests to return the spoils, e.g., “they have to give the stones back”), or *penalty* (responses involving every other kind of non-verbal punishment, e.g., “send them to prison,” “ban on watching TV,” “catch them with a trap”). Reliability (Cohen’s kappa) for all these measures together was excellent with $\kappa = .91$.

Accountability. We coded whether children thought that it would be okay if the transgressors knew about their tattling ("yes") or not okay ("no"). Reliability (Cohen's kappa) was excellent with $\kappa = 1$.

Exit choice and justification. For the exit choice, we assessed whether children wanted to leave their group or not ("yes" or "no"), and what reasons they gave for their choice. For their reasons, we coded the following categories: *transgression* (when children referred to the transgression, e.g., "because they took the gemstones," "because they didn't steal"), *policing* (when children wanted to stay in their group as a guard, e.g., "because I can tell you if they do something wrong," "I want to make sure that they will not steal again"), *group preference* (when children indicated that they identified with the group or preferred the group members in question, e.g., "I like the green group," "because I want to play with them"), *color preference* (when children referred to the group's color, e.g., "because green is my favorite color," "I like the yellow scarf better"), or *other* (any other statement that did not fall into one of the categories above, e.g., "because I want to"). Reliability (Cohen's kappa) was excellent with $\kappa = .91$.

Preference test. We assessed which puppet children gave the first marble to in the marble run game. If children gave this marble to one of their ingroup members, this was counted as preferring the ingroup, and if children gave this marble to one of the outgroup members, this was counted as preferring the outgroup. For children who had indicated that they wanted to change groups, we coded their preference according to their original group membership, i.e., the one they were allocated to at the beginning of the procedure. Reliability (Cohen's weighted kappa) was excellent with $\kappa = .92$.

Results

We did not push children to answer if they did not respond to the exploratory post-test questions. As a consequence the number of *no answer* responses was relatively high and the results should be taken with some caution. Thus, for most of the measures, statistical analysis was not appropriate due to small and uneven sample sizes in the different cells. In these cases we only report descriptive results.

Tattling words. We investigated whether children who tattled at some point ($n = 79$) used more negative words when tattling about the outgroup's transgression and/or the severe transgression. Table 1 shows the percentage of children who used each type of utterance in each condition. A Poisson-distributed GLM was run with group membership and transgression type as predictors. The full model did not differ from the null model ($p = .213$), indicating that there were no significant differences between conditions.

Transgression justifications. In response to the question about why the puppets had taken away the gemstones, children's relevant responses were mostly *positive/mitigating* (26%) or *neutral* responses (19%) overall. Table 1 shows the percentage of children who gave each response type in each condition. Any differences between conditions are difficult to interpret because of the small number of children who gave answers: overall, 50% of children did not answer this question.

Judgments and judgment justifications. Most children judged the transgression as either "not so good" or "bad." Only two children (both in the ingroup mild condition) judged the transgression as "good" or "ok". A Poisson-distributed GLM was run with group membership and transgression type as predictors for children's judgment score, but the full model did not differ from the null model ($p > .25$). Of the children who judged the transgression as being negative (i.e., either "not so good" or "bad"; all but two of the children), overall, 49% justified their responses with *normative* and 30% with *moral* reasons (see Table 1 for the justifications in each condition).

Punishment. Overall, 56% of children thought that the transgressors should be punished. Fourteen percent of all children suggested lecturing or scolding the transgressors verbally, 19% suggested that the transgressors be made to return the stones, and 25% proposed some sort of penalty. Strikingly, penalty, the most severe form of punishment, was rarely seen in the ingroup mild condition. Statistically there was no difference between conditions; the full-null GLM comparison was not significant ($p > .25$). However, again, because of high rates of *no answer* responses, this analysis should be interpreted with caution. Percentages for each condition are displayed in Table 1.

Accountability. Of the 79 children who tattled at some point, overall, 58% did not want the transgressors to know about their tattling, while 22% said it would be okay (20% gave no answer). Very few children in the ingroup mild condition wanted the transgressors to know about their tattling (see Table 1). However, statistically this difference between conditions was only marginal: The null model differed only marginally from the full model ($\chi^2(3) = 6.57, p = .087$). The full model revealed a marginal interaction effect (*Estimate* = -2.46, *SE* = 1.39, $\chi^2(1) = 3.67$, $p = .055$, Nagelkerke's $R^2 = .08$). Pairwise comparisons revealed marginal differences between the ingroup mild condition and all other conditions (Fisher's exact tests: $p = .067$, risk ratio = 6.07 with the ingroup severe condition; $p = .085$, risk ratio = 6 with the outgroup mild condition, and $p = .085$, risk ratio = 0.88 with outgroup severe condition).

Exit choice and exit choice justifications. When children's ingroup members were the transgressors, 46% of children wanted to leave their group after the mild transgression, but only 25% after the severe transgression. When the transgressors were outgroup members, 17% wanted to leave their group (and join the transgressor group) after the mild transgression, and 8% after the severe transgression (Table 1). Because children's tattling behavior might have influenced children's wish to change groups, we first tested whether children's exit choice was influenced by their tattling (yes/no), but found no relation between tattling and children's exit choice, GLM full vs. null model, $p >.25$). Then, a GLM was run to analyze the effect of condition on children's exit choice. The full model differed significantly from the null model ($\chi^2(3) = 10.22$, $p = .017$) and revealed no interaction between group membership and transgression type ($p > .25$). A main effect of group membership was found (*Estimate* = 1.39, *SE* = .54, $z = 2.57$, $p = .0091$, Nagelkerke's $R^2 = .11$), as well as a marginal main effect of transgression type ($p = .08$). Thus, children were more likely to leave their group when their group members had transgressed, and marginally less likely to leave their group after a severe transgression.

Overall, most children justified their response with their *color preference* (26%), a *group preference* (21%), the *transgression* (18%) or *other* reasons (19%). Only four percent of children justified their response with *policing*. Table 2 shows the percentage of children's reasons by condition. The transgression type did not seem to have a big impact on children's justification. We additionally looked at the data clustered in the following way: First, we were interested in what reasons children gave for *not wanting to be a member of the transgressors' group*, that is, why children in the ingroup condition wanted to leave the transgressors' group (n = 17), and why children in the outgroup condition did not want to join the transgressors' group (n = 42). Taken together, 29% of these children justified their decision with the *transgression* they had observed. Other common reasons were *color preference* (22%) and *group preference* (19%). Conversely, we were also interested in what reasons children gave for *wanting to be a member of the transgressors' group*, and therefore looked at the justifications of children in the ingroup condition who wanted to stay in the transgressors' group (n = 31) and children in the outgroup condition who wanted to join the transgressors' group (n = 6). Taken together, these children mostly explained their choice with *color preference* (32%) or *group preference* (24%). Interestingly, three of the children (8%) who wanted to stay in the transgressors' group justified this with *policing*, indicating that they would like to improve their group's behavior from within.

Preference test. Finally, we assessed children's ingroup preference by looking at whom they gave the first marble to when they were asked to allocate the marbles for the marble run game. In the ingroup condition, only 46% of children preferred their ingroup member in this task, compared to 69% of children in the outgroup condition who preferred their ingroup (see Table 1 for percentages by group and transgression conditions). To investigate the effect of condition on children's ingroup preference, we ran a GLM with group membership and transgression type as predictors for this binomial measure of ingroup preference. The null model differed only marginally from the full model ($\chi^2(3) = 6.65$, p = .084), and the interaction was not significant (p > .25). After removing the interaction from the model ($\chi^2(2) = 6.33$, p = .042), we found a main effect of group membership (*Estimate* = 0.97, *SE* = 0.43, z = 2.26, p = .024, Nagelkerke's R^2 = .07). Thus, children's ingroup preference was diminished when the transgression was conducted by their ingroup, compared to outgroup, members, while the severity of the transgression did not influence children's ingroup preference.

Tables

Table 1

For the Post-Test Measures, the Percentage of Children Giving each Type of Answer in each Group Membership and Transgression Type Condition

	Ingroup Conditions		Outgroup Conditions	
Measures	Mild	Severe	Mild	Severe
Tattling Words (*n* = 79)				
Negative	48%	69%	74%	61%
Neutral	52%	31%	26%	39%
Transgression Justifications (*n* = 96)				
Positive/Mitigating	33%	29%	17%	25%
Neutral	29%	13%	17%	17%
Negative/Aggravating	0%	4%	13%	4%
No answer	38%	54%	54%	54%
Judgments (*n* = 96)				
Good	4%	0%	0%	0%
Okay	4%	0%	0%	0%
Not so good	46%	46%	38%	29%
Bad	46%	42%	58%	67%
No answer	0%	13%	4%	5%
Judgment Justification (*n* = 94)				
Moral	23%	21%	33%	42%
Norm	50%	54%	58%	33%
Other	5%	0%	0%	4%
No answer	23%	25%	8%	21%
Punishment (*n* = 96)				
Yes	58%	54%	58%	54%
No	29%	29%	21%	21%
No answer	13%	17%	21%	25%
How to punish (*n* = 96)				
Lecturing	13%	17%	17%	8%
Compensating	29%	8%	13%	25%
Penalty	8%	25%	33%	33%
No answer	50%	50%	38%	33%
Accountability (*n* = 79)				
Yes	5%	31%	32%	22%
No	76%	56%	53%	48%
No answer	19%	13%	16%	30%
Exit Choice (*n* = 96)				
Leaving	46%	25%	17%	8%
Preference Test (*n* = 96)				
Ingroup Preference	38%	54%	67%	71%

Note. Justification judgments include only children who judged the transgression as negative (i.e., all but two children).

Table 2

Reasons Children in Each Group Membership and Transgression Type Condition Gave for Changing Groups or Staying in Their Group

	Ingroup Conditions		Outgroup Conditions	
Reasons	Mild	Severe	Mild	Severe
Changing (*n* = 23)				
Transgression	27%	33%	0%	0%
Policing	0%	0%	0%	0%
Group Preference	27%	17%	25%	50%
Color Preference	27%	17%	50%	50%
Other	9%	33%	0%	0%
No Answer	9%	0%	25%	0%
Staying (*n = 73*)				
Transgression	0%	0%	25%	32%
Policing	15%	6%	5%	0%
Group Preference	31%	17%	15%	18%
Color Preference	23%	33%	20%	23%
Other	23%	28%	20%	14%
No Answer	8%	17%	15%	14%
